# Predicting high-cost, commercially-insured people with diabetes in Texas: Characteristics, medical utilization patterns, and urban-rural comparisons

**DOI:** 10.3934/publichealth.2025016

**Published:** 2025-02-27

**Authors:** Lixian Zhong, Yidan Huyan, Elena Andreyeva, Matthew Lee Smith, Gang Han, Keri Carpenter, Samuel D Towne, Sagar N Jani, Veronica Averhart Preston, Marcia G. Ory

**Affiliations:** 1 Irma Lerma Rangel College of Pharmacy, Texas A&M University, College Station, TX, USA; 2 Center for Community Health and Aging, Texas A&M University, College Station, TX, USA; 3 Houston Methodist Academic Institute, Houston, TX, USA; 4 Department of Epidemiology and Biostatistics, School of Public Health, Texas A&M University, College Station, TX, USA; 5 Department of Health Policy and Management, School of Public Health, Texas A&M University, College Station, TX, USA; 6 Department of Health Behavior, School of Public Health, Texas A&M University, College Station, TX, USA; 7 Department of Environmental and Occupational Health, School of Public Health, Texas A&M University, College Station, TX, USA; 8 School of Global Health Management and Informatics, University of Central Florida, Orlando, FL, USA; 9 Disability, Aging, and Technology Cluster, University of Central Florida, Orlando, FL, USA; 10 Southwest Rural Health Research Center, Texas A&M University, College Station, TX, USA; 11 Health Care Service Corporation, Richardson, TX, USA

**Keywords:** T2DM, costs, healthcare utilization, commercially-insured, Texas

## Abstract

**Background:**

Type 2 diabetes mellitus (T2DM) is a prevalent chronic disease in the United States and healthcare resources used to manage the disease are disproportionately consumed by a small subset of users. Consequently, there is a potential to reduce the healthcare costs and to improve the health outcomes through the early detection and consistent management of high-cost users.

**Objective:**

The objectives of this study were to characterize the pattern of medical utilization and cost of commercially-insured people with type 2 diabetes (T2DM) in Texas and to identify predictors of high-cost users.

**Methods:**

Using claims data from a large commercial insurance plan spanning the period from 2016 to 2019, the total medical costs of a randomly selected 12-month period were analyzed for eligible commercially-insured people with T2DM, and the patients were categorized into the top 20% of high-cost users and the bottom 80% of lower-cost users. Descriptive analyses were conducted to describe the baseline characteristics of the people with T2DM, the patterns of healthcare utilization, and the costs of the two types of users. Multivariate logistic regression models were estimated to identify the predictors of being a high-cost T2DM user.

**Results:**

The top 20% of high-cost users accounted for 83% of the total medical cost, with an average cost of $41,370 as compared to only $2064 for the bottom 80% of lower-cost users. Several chronic conditions were identified to be strong predictors of being a high-cost patient. Rural high-cost users had, on average, fewer specialist visits but more inpatient stays compared to the urban high-cost users.

**Conclusion:**

Healthcare utilization and expenditures among commercially insured individuals with T2DM followed the 80–20 rule. High-cost users were strongly associated with worse health status. Residential rurality was not associated with high-cost use, though the patterns of resource utilization differed between urban and rural high-cost users.

## 1. Introduction

Healthcare resources in the United States (US) are disproportionately consumed by a small subset of the population, with a high demand for health management resulting in high healthcare costs. For example, the top 1% of healthcare utilizers account for over 20% of the total healthcare expenditures, while the bottom 50% only utilize 3% of the total healthcare expenditures [1]. Efforts focused on the early detection and consistent management of individuals with high healthcare cost have a great potential to reduce healthcare costs and improve the health outcomes [2].

Diabetes mellitus (DM) is a major chronic disease that impacts 10%–14% of the US population and is estimated to be associated with $412.9 billion in annual healthcare costs in 2022 [3]. On average, people with diabetes have a 2.6 times higher medical expenditure than those without diabetes, which is caused by complications ranging from elevated blood glucose levels to neurological, renal, peripheral vascular, cardiovascular, endocrine/metabolic, ophthalmic, and other end-organ damage [3]. Among the top 5% spenders, 37% (elderly patients) and 22% (non-elderly patients) had diabetes [4]. Although there has been extensive research about the costs associated with diabetes, few studies have focused on characterizing the high-cost healthcare users with diabetes [5],[6]. With the second largest population by state and a diabetes prevalence rate of 13% (i.e., one of the highest in the US) [7], the direct medical costs in Texas for diagnosed diabetes were 18.9 billion in 2017 [8]. In addition, 34% of the Texas adult population is considered pre-diabetic, which suggests that the disease burden will continue to grow in the coming years [8]. The purposes of this study are as follows: (1) to characterize the healthcare expenditure patterns of high-cost vs. lower-cost people with type 2 diabetes mellitus (T2DM) enrolled in a large commercial health plan; and (2) to identify predictors of high-cost healthcare users. Of special interest were factors associated with the costs in rural populations. Understanding the characteristics and utilization patterns of high-cost diabetes users can help policymakers, providers, payors, and other stakeholders make decisions regarding resource allocation to better manage the diabetes population, contain costs, and improve the health outcomes.

## 2. Materials and methods

### 2.1. Ethics approval of research

The Texas A&M Institutional Review Board (Organization Number: IORG00000397, IRB # IRB2020–0204) classified this study as “non-human subjects research” due to the absence of personal contact with the subjects and waived the need for ethical approval. Since the analysis involved non-experimental deidentified administrative claims data, obtaining informed consent from the study subjects and/or their legal guardians was not possible. All methodologies adhered to the applicable guidelines and regulations for a secondary data analysis.

### 2.2. Data source and patient selection

This analysis used claims data from a large commercial insurance plan with a significant presence in Texas between January 1^st^, 2016, and December 31^st^, 2019. The raw data was provided by the insurer in a longitudinal format aggregated by quarter. All beneficiaries aged 18–64 years, residing in Texas, with at least one medical claim of T2DM during the study period were included in the analysis. Anyone with a type 1 diabetes diagnosis in their claims during that period were excluded. Eligible study subjects were required to have at least 18 months of continuous enrollment during the study period, with no enrollment gap allowed. An index date (the first date of a quarter) was randomly selected during that period to ensure at least 12 months of post-index continuous enrollment to assess the cost and utilization and 6 months of pre-index continuous enrollment to assess the baseline characteristics post their first observed T2DM diagnosis.

#### 2.2.1 High-cost (all-cause) user definition

**Figure 1.**
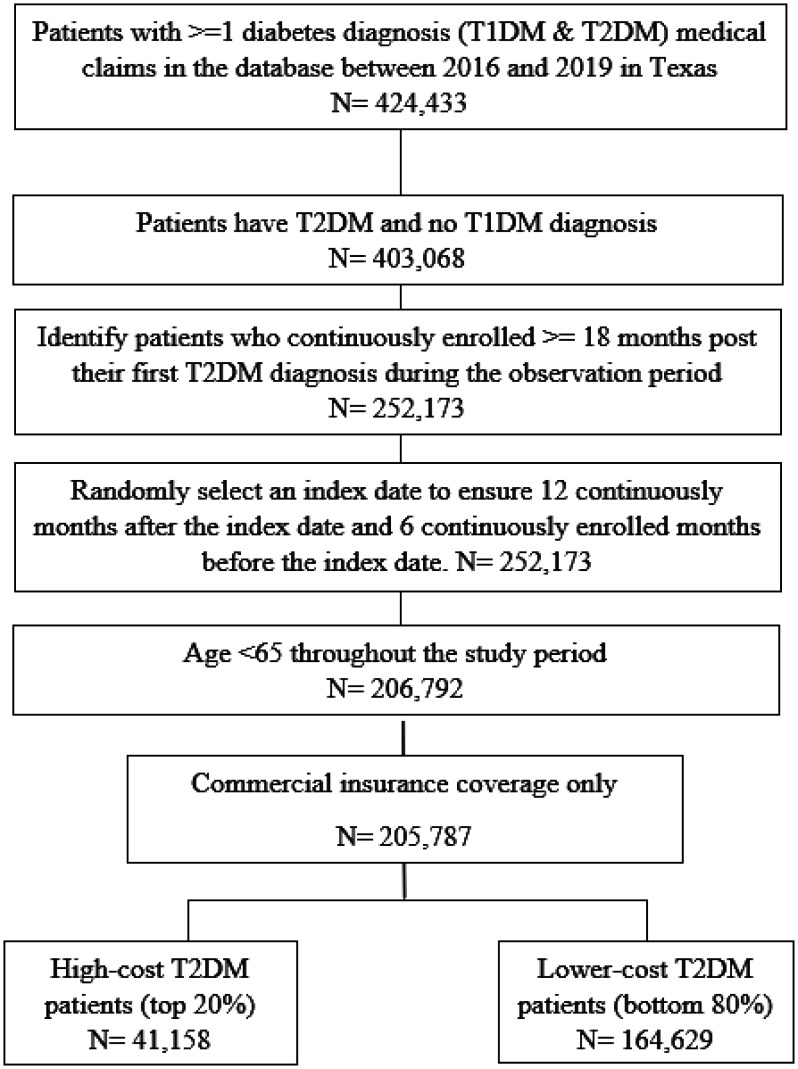
Sample selection.

Based on the total all-cause medical costs for the post-index 12-month period, which included inpatient, outpatient, and office visit costs, the patients were categorized into either the top 20% high-cost users or the bottom 80% lower-cost users (Figure 1). In addition, the total diabetes population was divided into five quintiles based on the total medical costs. The 1^st^ Quintile corresponds to the bottom 20% of the patient population, which had the lowest total medical costs, and the 5^th^ Quintile corresponds to the top 20% of the patient population, which had the highest total medical costs. The 5^th^ Quintile is equivalent to the definition of the top 20% high-cost users in this study.

### 2.3. Baseline patient characteristics

The baseline patient characteristics included demographics, insurance coverage status, general clinical information, and diabetes-related clinical information. The demographic characteristics of the patients, including age (18–34 years, 35–44 years, 45–54 years, and 55–64 years), sex (male vs. female), and rurality (urban vs. rural), were extracted from the index date. Rurality was measured based on the patient's county of residence, which was linked to a database maintained by the National Center for Health Statistics (NCHS) using the Urban-Rural Classification Scheme for Counties [9]. The NCHS Urban-Rural Classification Scheme includes 6 levels across metropolitan areas (large central metro, large fringe metro, medium metro, small metro) and non-metropolitan areas (micropolitan, non-core) [9]. In this study, these 6 categories were collapsed to create a binary variable to indicate the rurality: metropolitan /urban counties (large central metro, large fringe metro, medium metro, small metro) and non-metropolitan/rural counties (micropolitan, non-core).

The individuals who directly enrolled with the insurance plan or enrolled through an employer were recorded based on the index date enrollment status. The Charlson Comorbidity Index (CCI) (CCI score = 1, CCI score = 2 or 3 vs. CCI score ≥ 4) and a list of comorbidities defined by the International Classification of Diseases Version 10 (ICD 10) codes were evaluated in the 6-month period preceding the index date (pre-index period). The comorbidities included major comorbidities based on the CCI as well as the following diabetes-related comorbidities: acute myocardial infarction, arrhythmias, congestive heart failure, depression, moderate or severe renal disease, cerebrovascular disease (stroke), hemiplegia or paraplegia, atherosclerotic cardiovascular disease (ASCVD), dementia, chronic obstructive pulmonary disease (COPD), rheumatological disease, peptic ulcer disease, mild liver disease, moderate to severe liver disease, and metastatic solid tumor. Diabetes-specific characteristics were also assessed, which included short-term diabetes-related complications, long-term diabetes-related complications, and the presence of lower extremity amputation events during the pre-index period [10]. The HbA1c scores were only available in a subset of the identified people with T2DM (27.38%), and the average HbA1c score during the 6-month pre-index period was analyzed.

#### 2.3.1 Healthcare resources cost and utilization

The healthcare costs and utilization were evaluated through the 12-month post-index period. The measures of costs included the total medical cost and the individual cost categories including inpatient stays, outpatient visits, generalist visits, and specialist visits. Emergency Department (ED) visits were also reported, including ED outpatient visits (a subset of outpatient visits) and inpatient ED admissions (a subset of inpatient visits). All healthcare costs were inflated to 2019 U.S. dollars using the medical care component of the U.S. Consumer Price Index. Both the total costs of claims and the out-of-pocket (OOP) payments by beneficiaries were analyzed.

The utilization of medical resources was analyzed based on the same categories: inpatient stays, outpatient encounters, generalist visits, and specialist visits. The number of encounters were reported for different types of utilization for both the high-cost and the lower-cost users.

### 2.4. Statistical analysis

Descriptive analyses were conducted to describe the baseline characteristics of e the eligible study subjects, patterns of healthcare utilization, and the costs of the two types of users. The means and standard deviations were reported for continuous variables; the frequencies and percentages were reported for categorical variables. Two-mean independent t-tests were conducted for the continuous variables and chi-squared independence tests were performed for the categorical variables to record the statistical difference between the two groups (top 20% high-cost vs. bottom 80% lower-cost users).

A multivariate logistic regression model was estimated to identify the predictors of being a high-cost T2DM user. The dependent variable was a dichotomous variable (1 vs. 0) indicating whether the patient was in the high-cost user group or not. The independent variable was an indicator of being a high-cost diabetes patient (1/0). Other covariates included those baseline characteristics that revealed significant associations in the final model.

The results were considered statistically significant at p < 0.01 given the large sample size. All analyses were carried out using the Stata 14.2 statistical software (Stata®, College Station).

### 2.5. Subgroup analysis

Additional analyses were conducted to assess a subset of the population, namely the rural population, to characterize the baseline characteristics and to illuminate factors associated with the costs in the rural population using the same approach as described above.

## 3. Results

### 3.1. High-cost vs. Lower-cost user identification

Following the selection criteria, a sample of 205,787 people with T2DM were identified between 2016 and 2019 who were between the ages of 18 and 64 years and had a continuous enrollment with the Texas commercial insurer at least 6 months pre-index and 12 months post-index (Figure 1). Based on the all-cause total 12-month post-index medical cost, the top 20% high-cost and the bottom 80% lower-cost users were assigned. 41,158 users with an annual medical cost equal to or above $8490 were assigned to the top 20% high-cost user group, and they accounted for 83% of the total medical cost with an average cost of $41,370. The remaining 164,629 users with an annual medical cost below $8490 were assigned to the bottom 80% lower-cost user group and accounted for only 17% of the total medical cost, with an average cost of $2064 peruser, or only 1/20 of that in the top 20% users.

An additional cost analysis was performed by categorizing the overall medical cost into five quintiles. The distribution of costs by different types of utilization is illustrated in Supplementary Figure 1. In the fifth quintile, which is equivalent to the top 20% high-cost cohort, the inpatient cost is the major cost driver accounting for almost half of the total medical cost (49.5%), followed by outpatient visits (25.6%), specialist visits (20.8%), and generalist visits (3.9%). Notably, this top 20% accounts for 83% of the total medical costs, which is further broken down in each cost category: 99.75% of the total inpatient costs, 83.16% of the total outpatient costs, 75.48% of the total specialist visit costs, and 52.04% of the total generalist visit costs. The total costs in the other 4 quintiles were drastically lower, with each accounting for only 10.30% ($5110/patient), 4.00% ($1983/patient), 1.78% ($881/patient), and 0.57% ($282/patient) of the total medical cost, thus justifying the definitions of high-cost and low-cost users (Supplementary Table 1).

### 3.2. Baseline characteristics

The baseline characteristics of the high-cost and lower-cost users are summarized in Table 1. Relative to the lower-cost users, the high-cost users were more likely to be female (51.97% vs. 43.57%; p ≤ 0.001) and older (52.57 vs. 50.99; p ≤ 0.001). There were no significant differences between the two groups with regard to whether or not the beneficiary lived in an urban or a rural area (79.45% vs. 80.76%; p = 0.111).

The high-cost users generally had more comorbidities and higher CCI scores (score 2–3: 22.96% vs. 11.98%; score ≥ 4: 8.08% vs. 1.31%). The high-cost users had a significantly higher prevalence of all examined comorbidities (p ≤ 0.001), including arrhythmias (5.43% vs. 1.69%), congestive heart failure (2.93% vs. 0.55%), depression (4.24% vs. 1.66%), moderate or severe renal disease (4.24% vs. 1.66%), ASCVD (12.22% vs. 4.36%), rheumatological disease (2.78% vs. 0.79%), and mild liver disease (4.70% vs. 1.93%).

For diabetes-related clinical characteristics, the HbA1c values were only available in a small subset of the patients (27.62% of the high-cost vs. 27.31% of the lower-cost users had HbA1c values) and among the ones with baseline HbA1c data, there was no statistically significant difference between the two groups of users (7.310 vs. 7.321, p = 0.565). The high-cost users had increased rates of diabetes-related long-term complications (16.79% vs. 10.1%; p ≤ 0.001) and lower extremity amputations (0.11% vs. 0.01%; p ≤ 0.001). However, there was no statistically significant difference in the rates of short-term complications between the two groups (19.72% vs. 17.44%; p = 0.209).

### 3.3. Costs and utilization

The costs and utilization for the high-cost and lower-cost users are summarized in Table 2. The all-cause average annual total medical cost of high-cost users was $41,370 (median: $19,794), of which 38.61% ($15,973) was attributed to inpatient costs ($6993 were through ED), 30.86% ($12,767) to outpatient costs ($3057 were through ED), and 30.53% ($12,630) to professional visit costs, with $2562 for generalist visits and $10,068 for specialist visits. The average high-cost users' all-cause medical costs were nearly 20 times higher ($41,370 vs. $2064). The average out-of-pocket (OOP) medical expense among the high-cost patients was 5 times higher than that of the lower-cost users ($3918 vs. $712).

Additionally, the high-cost users had a higher number of visits and a longer inpatient length of stay than the lower-cost users. Notably, 99.75% of the inpatient costs, 83.16% of the outpatient costs, and 75.38% of the specialist costs were incurred by the high-cost user group (Supplementary Figure 1). In addition, all-cause specialist visits accounted for the largest number (32.8 visits/person/year) of visits among the high-cost users compared to 6.0 visits/person/year among the lower-cost patients.

### 3.4. Regression analysis results

The multivariate regression model (Table 3) identifies predictors of being a high-cost user. In general, female gender (OR = 1.41; p ≤ 0.0001), older age (OR = 1.13; p ≤ 0.0001), and individuals with more comorbidities (OR = 1.79 for CCI = 2/3 vs. 1, OR = 2.94 for CCI ≥ 4 vs. 1; p ≤ 0.0001) were associated with increased odds of being a high-cost user. The strongest predictor was being on dialysis (OR = 68.16; p ≤ 0.0001), followed by the diagnosis of a metastatic solid tumor (OR = 7.75, p ≤ 0.0001) (Table 3). All other listed chronic comorbidities were found to be associated with significantly increased odds of being a high-cost user. Notably, dementia (OR = 3.16; p ≤ 0.0001), moderate or severe liver disease (OR = 2.72; p ≤ 0.0001), depression (OR = 2.42; p ≤ 0.0001), and hemiplegia/paraplegia (OR = 2.29; p ≤ 0.0001) were found to be associated with relatively high odds of being a high-cost user. In addition, using a pump or having lower extremity amputations was associated with a significantly higher risk of being a high-cost user (OR 4.43 and 4.23 respectively; p ≤ 0.0001).

**Table 1.** Baseline characteristics, stratified by total health care cost cohort.

	Top 20% High-Cost Users n = 41,158		Botton 80% Lower-Cost Users n = 164,629		p-value

	N or Mean	% or SD	N or Mean	% or SD	
Female	21,389	51.97%	71,722	43.57%	<0.001
Age	52.27	9.075	50.99	9.333	<0.001
18–34	2161	5.25%	10,309	6.26%	<0.001
35–44	5574	13.54%	27,274	16.57%	
45–54	12,975	31.52%	56,390	34.25%	
55–64	20,448	49.68%	70,656	42.92%	
Urban	32,701	79.45%	132,958	80.76%	0.111
Rural	5781	14.05%	22,914	13.02%	
Commercial fully insured coverage	17,326	42.10%	70,016	42.53%	0.112
CCI index					
1	28,382	68.96%	142,751	86.71%	<0.001
2 or 3	9449	22.96%	19,722	11.98%	
≥4	3327	8.08%	2156	1.31%	

Comorbidities					
Acute Myocardial Infarction	340	0.83%	329	0.20%	<0.001
Arrhythmias	2234	5.43%	2785	1.69%	<0.001
Congestive Heart Failure	1205	2.93%	900	0.55%	<0.001
Depression	1744	4.24%	2738	1.66%	<0.001
Opioid Use Disorder	203	0.49%	217	0.13%	<0.001
Moderate or Severe Renal Disease	2434	5.91%	1917	1.16%	<0.001
Cerebrovascular Disease (Stroke)	477	1.16%	432	0.26%	<0.001
Hemiplegia or Paraplegia	156	0.38%	78	0.05%	<0.001
ASCVD§	5030	12.22%	7174	4.36%	<0.001
Dementia	131	0.32%	92	0.06%	<0.001
Chronic Obstructive Pulmonary Disease	859	2.09%	892	0.54%	<0.001
Rheumatological Disease	1146	2.78%	1294	0.79%	<0.001
Peptic Ulcer Disease	156	0.38%	183	0.11%	<0.001
Mild Liver Disease	1934	4.70%	3178	1.93%	<0.001
Moderate to Severe Liver Disease	229	0.56%	92	0.06%	<0.001
Metastatic Solid Tumor	427	1.04%	73	0.04%	<0.001
Kidney failure (on dialysis)	968	2.35%	19	0.01%	<0.001

Diabetes-related characteristics					
HbA1c level (pre-index)*	7.310 (11,369)	27.62%	7.321 (44,966)	27.31%	0.565
Short-term complications	8116	19.72%	28,717	17.44%	0.209
Long-term complications	6909	16.79%	16,483	10.01%	<0.001
Lower extremity amputations	46	0.11%	14	0.01%	<0.001
Pump use	546	1.33%	364	0.22%	<0.001

Note: *Only a subset of study subjects had HbA1c data. Results were considered statistically significant at p < 0.01 given the large sample size. §ASCVD: Atherosclerotic cardiovascular disease.

**Table 2.** Health care utilization and costs in the 12-month observational period.

	Top 20% High-cost Users			Bottom 80% Lower-cost Users		

	Mean (non-zero mean*)	Median (Proportion**)	SD	Mean (non-zero mean*)	Median (Proportion**)	SD
Total medical costⱡ	$41,370	$19,794 (1)	$74,139	$2064	$1275 (0.96)	$2032
Total OOP medical cost	$3918	$3378 (0.995)	$3775	$712	$354 (0.94)	$914
Inpatient						
Length of stay	1.82 (4.83)	0 (0.38, 3)§	4.12	0.0064 (2.65)	0 (0.0024, 2)§	0.16
Number of visits	0.59 (1.56)	0 (0.38)	1.1	0.0025 (1.03)	0 (0.0024)	0.05
Cost	$15,973 ($42,414)	$0 (0.38)	$55,307	$10 ($4074)	$0 (0.0024)	$216
OOP cost	$677 ($2592)	$0 (0.26)	$2212	$4 ($1859)	$0 (0.0022)	$106
ED-inpatient					
Number of visits	0.31 (1.35)	0 (0.23)	0.73	0 (1)	0 (0.0015)	0.04
Cost	$6993 ($30,763)	$0 (0.23)	$28,781	$7 ($4443)	$0 (0.0015)	$182
OOP cost	$397 ($2573)	$0 (0.15)	$1595	$3 ($2082)	$0 (0.0014)	$94
Outpatient
Number of visits	7.12	4	12.48	0.92 (2.11)	0 (0.43)	1.63
Cost	$12,767	$6703	$28,107	$646 ($1489)	$0 (0.43)	$1188
OOP cost	$1521	$948	$2379	$228 ($783)	$0 (0.29)	$565
ED-outpatient
Number of visits	1.08	1	1.98	0.16 (1.23)	0 (0.13)	0.46
Cost	$3057	$396	$6170	$259 ($2016)	$0 (0.13)	$838
OOP cost	$614 ($1518)	$0 (0.40)	$1230	$119 ($976)	$0 (0.12)	$439
Generalist visits
Number of visits	12.04	9	14.65	4.17	4	3.57
Cost	$2562	$1565	$4968	$590	$451	$573
OOP cost	$1126	$760	$1310	$615	$404	$688
Specialist visits
Number of visits	32.77	23	37.77	6	4	6.61
Cost	$10,068	$5445	$18,990	$817	$385	$1091
OOP cost	$1233	$831	$1450	$295	$111	$477

Note: *Means of non-zero values were calculated for those with median = 0; **Proportions of non-zero values were calculated for those with median = 0; §For length of stay, the proportions of non-zero values and the median for non-zero values were included in (); ⱡTotal medical cost was calculated as the sum of inpatient, outpatient, generalist, and specialist costs. ED-inpatient cost was a subset of total inpatient cost and ED-outpatient cost was a subset of total outpatient cost.

**Table 3.** Multivariate regression model.

	Odds ratio	Lower 95% CI	UUpper 95% CI	p-value
Baseline characteristics				
Female (vs. Male)	1.41	1.37	1.45	<0.001
Age 55–64 (vs. <55)	1.13	1.09	1.16	<0.001
Commercial fully insured coverage (vs. no commercial fully insured coverage)	0.93	0.91	0.96	<0.001

CCI (vs. CCI = 1)				
2 or 3	1.80	1.74	1.86	<0.001
≥4	2.95	2.74	3.19	<0.001

Comorbidities (vs. no such comorbidity)				
Arrhythmias	1.89	1.76	2.03	<0.001
Congestive Heart Failure	1.89	1.71	2.09	<0.001
Depression	2.42	2.24	2.62	<0.001
Moderate or Severe Renal Disease	1.40	1.29	1.52	<0.001
Hemiplegia or Paraplegia	2.52	1.87	3.40	<0.001
ASCVD§	1.93	1.84	2.02	<0.001
Dementia	3.18	2.29	4.42	<0.001
Chronic Obstructive Pulmonary Disease	2.05	1.85	2.28	<0.001
Rheumatological Disease	2.28	2.10	2.49	<0.001
Mild Liver Disease	1.48	1.39	1.58	<0.001
Moderate to Severe Liver Disease	2.71	2.09	3.53	<0.001
Metastatic Solid Tumor	7.72	5.94	10.04	<0.001
Kidney failure (on dialysis)	68.16	42.97	108.11	<0.001

Diabetes-related characteristics				
Short-term complications (vs. no short-term complications)	1.05	1.02	1.08	0.002
Lower extremity amputations (vs. no lower extremity amputations)	4.21	2.18	8.15	<0.001
Pump use (vs. no pump use)	4.43	3.76	5.22	<0.001

Note: Results were considered statistically significant at p < 0.01 given the large sample size. §ASCVD: Atherosclerotic cardiovascular disease.

### 3.5. Subgroup analyses on rural population

Subgroup analyses were conducted to compare the healthcare utilization and costs of the commercially insured rural population versus the urban population (Table 4). The subgroup analyses showed that there was no statistically significant association between rurality and being in the high-cost/lower cost cohorts. The overall patient population had 14.76% residing in rural areas as compared to 15.02% for the high-cost users (p = 0.111). Among the high-cost users, while the rural enrollees were on average not statistically different in total medical cost compared to the urban enrollees ($40,716 vs. $40,846; p = 0.9016), they exhibited slightly different utilization patterns. The rural high-cost enrollees had more inpatient stays (0.62 vs. 0.57; p = 0.0028) and more outpatient visits (7.69 vs. 6.92, p ≤ 0.000), including more ED outpatient visits (1.23 vs. 1.04; p ≤ 0.0001). However, rural high-cost users had significantly fewer specialist visits compared to the urban enrollees (29.32 vs. 32.95; p ≤ 0.0001). The statistics on healthcare utilization and the costs for the rural population are summarized in Supplementary Table 2. The multivariate regressions used to identify the predictors for the high-cost rural populations are summarized in Supplementary Table 3. The predictors for the high-cost users generally hold for the rural population as well.

**Table 4.** Comparison of healthcare utilization and costs among high-cost users in rural vs. urban areas.

Top 20% high-cost users					
	Rural (n = 5781)		Urban (n = 32,701)		p-value

	Mean	SE	Mean	SE	
Total medical cost	$40,716	$940	$40,846	$408	0.9016
Total OOP medical cost	$3880	$38	$3936	$22	0.2996
Inpatient					
Length of stay	1.97 (4.96, 3)§	4.20	1.76 (4.76, 3)§	4.05	0.0003
Number of visits	0.62	1.08	0.57	1.08	0.0028
Cost	$14,895	$49,951	$15,775	$55,778	0.2619
OOP cost	$628	$1479	$690	$2365	0.0541
ED-inpatient					
Number of visits	0.28	0.64	0.31	0.73	0.0037
Cost	$5265	$21,992	$7.108	$29,520	<0.0001
OOP cost	$327	$1095	$411	$1697	0.0003
Outpatient					
Number of visits	7.69	10.49	6.92	12.35	<0.001
Cost	$13,738	$30,494	$12,484	$27,504	0.0017
OOP cost	$1647	$1857	$1502	$2422	<0.0001
ED-outpatient					
Number of visits	1.23	2.05	1.04	1.94	<0.0001
Cost	$3046	$5874	$3028	$6177	0.8393
OOP cost	$632	$1186	$613	$1245	0.2919
Generalist visits					
Number of visits	12.01	12.26	11.93	14.52	0.6738
Cost	$2323	$5111	$2583	$4849	0.0002
OOP cost	$1101	$1234	$1162	$1345	0.0015
Specialist visits					
Number of visits	29.32	30.93	32.95	38.04	<0.0001
Cost	$9760	$23,366	$10,005	$18,019	0.3653
OOP cost	$1149	$1341	$1249	$1465	<0.0001

Note: §For length of stay, the mean and median for non-zero values were included in (). Results were considered statistically significant at p < 0.01 given the large sample size.

## 4. Discussion

This study examined healthcare expenditure patterns of high-cost vs. lower-cost users with T2DM enrolled in a large commercial health plan in Texas and identified predictors of high medical costs. The high-cost individuals were more likely to be older and had more comorbidities. They utilized more health services overall, particularly for outpatient encounters and inpatient stays. Subgroup analyses revealed that the rural high-cost users tended to have fewer specialist visits, but more ED visits compared to their urban counterparts.

To our knowledge, this is the first study to describe patterns of healthcare utilization and expenditures among high-cost utilizers diagnosed with T2DM using administrative claims data for commercially-insured individuals in the state of Texas. Commercially-insured people with T2DM differ from Medicaid, Medicare, or uninsured populations in terms of age, socioeconomic status, and access to care, among other factors. Additionally, approximately 40% of the Texas population is of Hispanic origin, which is higher than in most other parts of the US. Generally, the Hispanic population exhibits a higher prevalence of diabetes and obesity as compared to the non-Hispanic White population, which may influence the observed healthcare utilization patterns [11]–[13]. Our study illustrates that care utilization and expenditures among people diagnosed with T2DM in this population are highly skewed, with the top 20% high-cost users consuming over 80% of all healthcare services, thus confirming the oft-cited 80/20 healthcare rule [14], where a small proportion of healthcare users consumes a disproportionate amount of health care dollars.

The high-cost users might benefit from healthcare models that encourage disease monitoring, management and care coordination, such as community-oriented programs [15]–[17]. Our findings support a recent trend among commercial insurers to use a more holistic approach in managing one's treatment, with a particular focus on the overall wellbeing, especially for older adults with multiple chronic comorbidities [18]–[20]. This trend is especially prevalent in Medicare Advantage plans, many of which now offer supplemental benefits, such as healthy foods and transportation. These supplemental benefits often address social determinants of health (SDoH) to help patients manage their healthcare needs [21]. Access to healthy foods is particularly important for people with diabetes, which is a metabolic disease. Commercial plans are encouraged to consider adopting these strategies in managing people with T2DM. Continuous management and timely interventions help people with diabetes stay in control of their disease and can slow disease progression and reduce the development of acute and chronic complications. In the long run, this will help prevent their healthcare expenditures from spiraling out of control [20],[22]–[24].

While it is important to manage the high-demand, high-cost individuals with T2DM to curb the overall medical cost, it is also critical to manage the early-stage, low-cost patients so that their diseases do not progress into more severe cases that require more healthcare utilization. These include, but are not limited to, routine screening, monitoring, and healthy lifestyle education. It is recommended that people with diabetes who take oral pills or those who manage their diseases through diet alone should see a doctor at least every 4–6 months. Our data suggest that most lower-cost users have their diseases monitored regularly, with a median of 2 for generalist visits and 1 for specialist visits that are related to their diabetes diagnosis (data not shown). However, the high skewness of the utilization data also suggested that the bottom 20%–40% percentile of people with diabetes may not be receiving their optimal care. Future studies should characterize the under-utilizers and investigate their unmet healthcare needs.

This study found that, in the high-cost cohort, although the urban and rural enrollees, on average, had similar medical costs, their healthcare utilization patterns were slightly different. The rural population had more inpatient stays, more outpatient visits, but fewer specialty care visits. Access to specialty care may be an SDoH barrier to some people residing in rural Texas and presents an opportunity for care improvement. The increased hospitalizations may reflect poor disease management outcomes, and a previous study showed that preventable hospitalizations were associated with a lack of specialty care in rural areas [25]. Increasing access to new models of care delivery, such as telehealth, should be explored and implemented to improve access to specialty care in these areas [26]. These changes may lead to improved health outcomes and ultimately reduce healthcare costs with reduced hospitalizations.

### 4.1. Limitations

This study has several limitations. First, data about the prescription medication utilization and costs were not available for this study. Therefore, we only examined medical costs without including pharmacy costs. Second, race and ethnicity may be associated with the healthcare utilization. However, the race and ethnicity information was not available in these data; therefore, it was not possible to control for race/ethnicity in the predictive regression model. Third, blood glucose control is critical in the care of people with type 2 diabetes. However, the HbA1c values were only available for about a quarter of the enrollees with diabetes who had their lab values linked to the claims data. The baseline comparison showed that the HbA1c values were not statistically different (7.310 vs. 7.321, p = 0.565), and thus it was not included in the final regression model. In a separate regression model including HbA1c, it was not associated with the odds of being a high-cost patient (data not shown). While this is counterintuitive, it suggests that the HbA1c data may not be missing at random in this dataset. Finally, while the administrative claims data offers a snapshot of health care utilization and costs for a large population base, this data set does not permit assessment of when patients were first diagnosed with specific conditions, thus limiting the conclusions about the causality of different predictors.

## 5. Conclusions

Healthcare utilization and expenditures among commercially-insured individuals with T2DM were highly skewed following the 80–20 rule. The high-cost users were strongly associated with a worse health status. Residential rurality did not appear to be associated with being a high-cost user, though the underlying resource utilization patterns differed between the urban and rural high-cost diabetes users.

## Use of AI tools declaration

The authors declare they have not used Artificial Intelligence (AI) tools in the creation of this article.


