# Supplementary material for: Predicting high-cost, commercially-insured people with diabetes in Texas: Characteristics, medical utilization patterns, and urban-rural comparisons
Source: AIMS Public Health. 2025 Feb 27;12(1):259–74. doi: 10.3934/publichealth.2025016 (PMC11999814; doi:10.3934/publichealth.2025016)
Supplement: Supplementary file 1 [file publichealth-12-01-016-s001.pdf]

Public Health

AIMS Public Health, 12(1): 259–274.
DOI: 10.3934/publichealth.2025016
Received: 31 May 2024
Revised: 06 December 2024
Accepted: 30 December 2024
Published: 27 February 2025

https://www.aimspress.com/journal/aimsph

Research article

Predicting high-cost, commercially-insured people with diabetes in Texas: Characteristics, medical utilization patterns, and urban-rural comparisons

Lixian Zhong[1,2,3,*], Yidan Huyan[4], Elena Andreyeva[5], Matthew Lee Smith[2,6], Gang Han[2,4], Keri Carpenter[2], Samuel D Towne Jr.[2,7,8,9,10], Sagar N Jani[2], Veronica Averhart Preston[11,§] and Marcia G. Ory[2,7]

[1] Irma Lerma Rangel College of Pharmacy, Texas A&M University, College Station, TX, USA
[2] Center for Community Health and Aging, Texas A&M University, College Station, TX, USA
[3] Houston Methodist Academic Institute, Houston, TX, USA
[4] Department of Epidemiology and Biostatistics, School of Public Health, Texas A&M University, College Station, TX, USA
[5] Department of Health Policy and Management, School of Public Health, Texas A&M University, College Station, TX, USA
[6] Department of Health Behavior, School of Public Health, Texas A&M University, College Station, TX, USA
[7] Department of Environmental and Occupational Health, School of Public Health, Texas A&M University, College Station, TX, USA
[8] School of Global Health Management and Informatics, University of Central Florida, Orlando, FL, USA
[9] Disability, Aging, and Technology Cluster, University of Central Florida, Orlando, FL, USA
[10] Southwest Rural Health Research Center, Texas A&M University, College Station, TX, USA
[11] Health Care Service Corporation, Richardson, TX, USA

* **Correspondence:** Email: zhong@tamu.edu; Tel: +19794360193.

§ Dr. Preston was employed at Health Care Service Corporation at the time of the research but is no longer with the company.

Supplementary

Table 1. Overall health care costs during the observational period.

Overall					
Mean	1st quintile (0%–20%)	2nd quintile (20%–40%)	3rd quintile (40%–60%)	4th quintile (60%–80%)	5th quintile (80%–100%)
Total	$281.56	$881.31	$1982.85	$5110.08	$41,369.76
Inpatient	$0.00	$0.04	$0.33	$39.13	$15,973.27
Outpatient	$10.73	$87.21	$433.38	$2053.92	$12,767.02
Generalist	$180.57	$467.90	$692.04	$1020.33	$2561.97
Specialist	$90.25	$326.17	$857.12	$1996.70	$10,068.30

Table 2. Health care utilization and costs in the 12-month observational period in rural population.

	Top 20% (n = 5781)			Bottom 80% (n = 22,914)		
	Mean (%)	Median	SD	Mean (%)	Median	SD
Total medical cost	$40,716	$19,877	$71,471	$2045	$1238	$2052
Total OOP medical cost	$3880	$3482	$2873	$733	$358	$960
Inpatient						
Length of stay	3.74	0	12.48	0.01	0	0.27
Number of visits	0.62	0	1.08	0.005	0	0.07
Cost	$14,895	$0	$49,951	$18.20	$0	$283
OOP cost	$628	$0	$1479	$8	$0	$156
ED-inpatient						
Number of visits	0.28	0	0.64	0.003	0	0.05
Cost	$5265	$0	$21,992	$11	$0	$224
OOP cost	$327	$0	$1095	$6	$0	$132
Outpatient						
Number of visits	7.69	5	10.49	1.41	1	1.98
Cost	$13,738	$7287	$30,494	$804	$172	$1277
OOP cost	$1647	$1109	$1857	$289	$0	$615
ED-outpatient						
Number of visits	1.23	1	2.05	0.21	0	0.55
Cost	$3046	$823	$5874	$285	$0	$846
OOP cost	$632	$0	$1186	$132	$0	$447
Generalist visits						
Number of visits	12.01	9	12.26	4.26	4	3.56
Cost	$2323	$1420	$5111	$541	$404	$544
OOP cost	$1101	$753	$1234	$618	$407	$680
Specialist visits						
Number of visits	29.32	21	30.93	5.31	3	6.07
Cost	$9760	$4891	$23,366	$683	$286	$988
OOP cost	$1149	$757	$1341	$254	$83	$445

Note: Results were considered statistically significant at P < 0.01 given the large sample size.

Table 3. Multivariate Regression Model to identify predictors for high-cost patients in rural areas.

Variables	Odds ratio	Lower 95% CI	Upper 95% CI	*P*
Patient characteristics				
Female (vs. Male)	1.32	1.22	1.42	0.000
Commercial fully insured coverage (vs. no commercial fully insured coverage)	0.84	0.78	0.90	0.000
CCI (vs. CCI = 1)				
2 or 3	1.94	1.78	2.12	0.000
≥4	3.51	2.87	4.30	0.000
Comorbidities (vs. no such comorbidity)				
Arrhythmias	1.77	1.48	2.13	0.000
Congestive Heart Failure	1.85	1.47	2.32	0.000
Depression	2.16	1.69	2.78	0.000
Moderate or Severe Renal Disease	1.34	1.08	1.65	0.008
With ASCVD	2.24	1.99	2.51	0.000
Chronic Obstructive Pulmonary Disease	1.47	1.17	1.85	0.001
Rheumatological Disease	2.21	1.76	2.78	0.000
Mild Liver Disease	1.87	1.57	2.22	0.000
Metastatic Solid Tumor	5.17	2.79	9.59	0.000
Kidney failure (on dialysis)	25.63	7.89	83.29	0.000
Diabetes characteristics				
Pump use (vs. no pump use)	4.62	2.87	7.44	0.000

Note: Results were considered statistically significant at P< 0.01 given the large sample size.

Figure 1. Costs for different types of health care utilization by quintile.

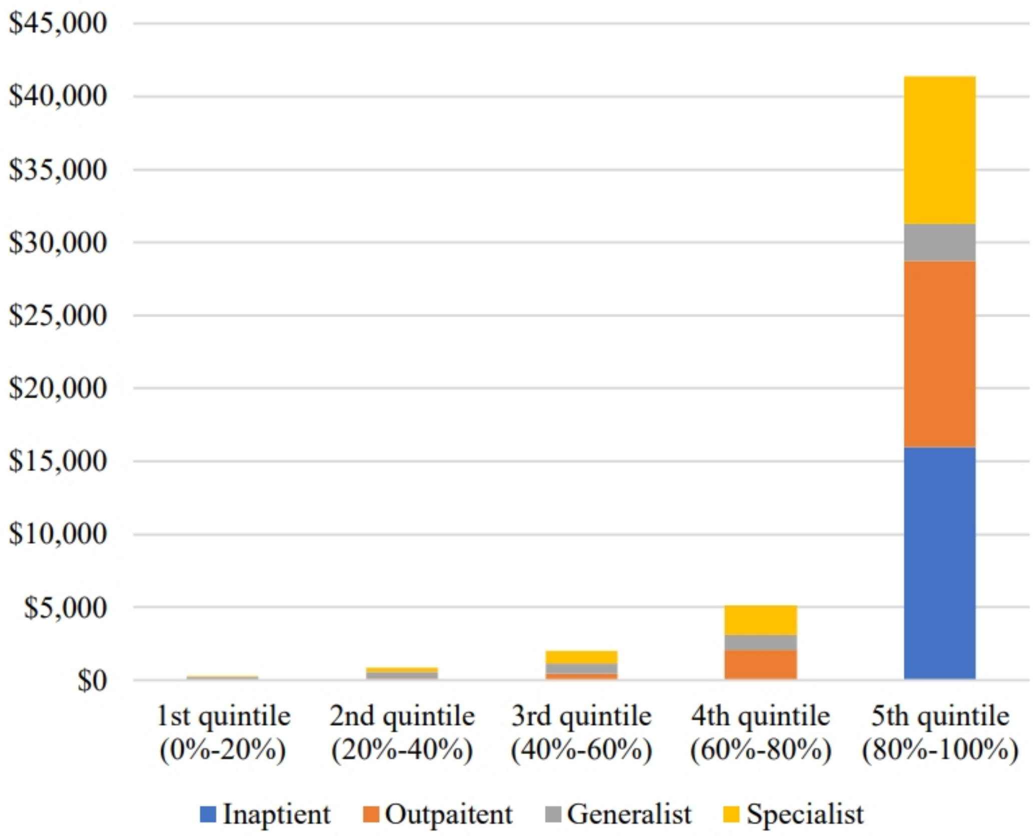

AIMS Press

© 2025 the Author(s), licensee AIMS Press. This is an open access article distributed under the terms of the Creative Commons Attribution License (https://creativecommons.org/licenses/by/4.0)
